# Prevalence and associated factors of childhood overweight/obesity among primary school children in urban Nepal

**DOI:** 10.1186/s12889-019-7406-9

**Published:** 2019-08-06

**Authors:** Ashmita Karki, Archana Shrestha, Narayan Subedi

**Affiliations:** 1 Department of Community Medicine and Public Health, Institute of Medicine, Tribhuvan University, Kathmandu, Nepal; 2 Department of Community Programs, Kathmandu University School of Medical Sciences, Dhulikhel, Nepal; 3 Department of Epidemiology, Harvard T H Chan School of Public Health, Boston, MA USA

**Keywords:** Overweight/obesity, Body mass index, Prevalence, Children, Nepal

## Abstract

**Background:**

Childhood overweight/obesity has become a major public health concern globally because of its adverse health consequences and escalating prevalence. The factors underlying the disease conditions manifested during adulthood commonly originate in childhood. Nepal is going through a transition where under-nutrition co-exists with obesity; however, there is a lack of well-documented information on childhood overweight or obesity in Nepal. This study was carried out to determine the prevalence and associated factors of childhood overweight/obesity among urban primary school children.

**Methods:**

A cross-sectional survey was conducted from May to October of 2017. Behavioral data were collected using a structured self-administered questionnaire with parents of children aged 6–13 years old in grades 1–5 studying in private schools of Lalitpur district in Nepal. Study participants were selected using two-stage cluster random sampling from 10 private schools. Height and weight measurements of 575 children were taken and BMI-for-age-sex was calculated using WHO AnthroPlus. Data were analyzed using SPSS version 21. Associated factors were examined using Chi-square tests followed by multivariate logistic regression analyses.

**Results:**

The study found that out of 575 students, 107 (18.6%) were overweight and 41 (7.1%) were obese. Among 328 male children, 62 (19.0%) were overweight and 35 (10.6%) were obese. Likewise, among 247 female children, 45 (18.2%) were overweight and 6 (2.4%) were obese. Male children (aOR = 2.21, 95% CI: 1.38–3.53), children of mothers with a high school (aOR = 3.13, 95% CI: 1.39–7.12) or university level of education (aOR = 3.09, 95% CI: 1.23–7.70) and children of mothers in a professional field (aOR = 1.34, 95% CI: 1.02–4.05) had a greater likelihood of being overweight/obese. Likewise, students consuming energy-dense less nutrient food (aOR = 2.92, 95% CI: 1.66–5.12), lacking active travel to and from school (aOR = 2.38, 95% CI: 1.12–4.79) and those having sedentary behaviors (aOR = 3.01, 95% CI: 1.20–7.29) were likely to be overweight/obese.

**Conclusions:**

More than one-quarter of the children in urban Lalitpur were found to be overweight/obese. High junk food consumption and sedentary activity were found to be significantly associated with childhood overweight/obesity. School health and awareness programs aiming to reduce the intake of energy-dense foods and promote an active lifestyle including active transportation to school among children are imperative. Future studies to objectively measure the type and amount of food intake and physical activity of students are recommended.

## Background

Overweight and obesity now rank as the fifth leading risk for mortality worldwide [1]. Although the health consequences of obesity are mostly manifested during adulthood, the factors underlying the disease’s conditions commonly originate during childhood [2]. Overweight and obese children are more likely to grow to become overweight and obese adults with higher chances of developing non-communicable diseases like diabetes and CVDs. Once called a high-income country problem, the rate of increase of childhood overweight and obesity is 30% higher in low and middle-income countries than in high-income nations [3–5]. The prevalence of childhood obesity in such nations has increased by 28% in just a couple of years [6]. There were 12.4 million obese children in Asia alone in 1990 (1.2 million in Southeast Asia), which increased to 18 million in 2010 (2.5 million in Southeast Asia). If this trend continues, there will be 24 million obese Asian children by 2020 [5–8].

Nepal is going through a transition where nutrition deficit coexists with obesity [9]. Although the prevalence of under-nutrition is higher than over-nutrition, this coexistence may give rise to what is known as “the double burden of malnutrition” very soon. The Nepal Demographic Health Survey (NDHS) 2016 has revealed that 1% of the children under-five years of age are overweight in Nepal [9]. However, no national data exist on the prevalence of overweight among children above 5 years of age.

Childhood obesity affects all, irrespective of their age, sex, or ethnicity. However, it is found to be particularly prevalent in areas that have undergone economic growth, urbanization, [10–12] technological advancement, and food behavior modification [13–17], which is a similar characteristic in urban Nepal. Yet, there are few studies reporting childhood obesity and its risk factors in Nepal. A study from Kaski district in Nepal found that the odds of having overweight/obese children in urban households were 2.3 times higher compared to rural households (*p* = 0.001, OR = 2.3) [18]. Previous studies in Nepal have mainly focused on socio-demographic factors related to childhood overweight/obesity (OW/OB) and none reported diet and physical activity-related risk factors [2, 10, 18–20]. Children should be considered the priority population for intervention since it is difficult to reduce excessive weight once it gets established [21].

This study aims to assess the prevalence and factors associated with childhood overweight and obesity among 6–13 year old children of an urban area of Lalitpur district in central Nepal. The study results could be useful for health managers and other stakeholders to plan prevention programs for childhood obesity in Nepal with a similar context.

## Methods

### Study setting

The study was conducted in private schools of Lalitpur Metropolitan City in central Nepal. The city was selected purposively, as more than 80% of private schools in Lalitpur are clustered there. A total of 125 private schools operate up to grade 5 or higher in this area.

### Study population

The study population was the primary level (grade 1–5) school children of private schools, aged 6–13 years. The respondents were any of the parents of the child. Children with amputated body parts, or any acute or chronic health condition were excluded from the study as these conditions could affect their body weight. Those staying in a hostel away from their parents were also excluded as the questionnaire had to be filled out by a parent.

### Study design and sample

This was a cross-sectional descriptive study conducted in May–October 2017. We obtained the list of private schools from Lalitpur District Education Office (DEO), Lalitpur. Two-stage cluster random sampling was performed. In the first stage, we randomly selected 10 out of the total 125 schools. In the second stage, we randomly selected two classes between grades 1–5 in each school and enrolled all students. Each class averaged about 30 students.

Since similar studies have not been carried out in Nepal to base our sample estimation upon, prevalence was assumed to be 50% [22] and sample size was calculated using formula; n = z2pq/e2 where *p* = 0.5, q = 0.5, z = 1.96 at 95% confidence interval, e = allowable error of 11% of p. Assuming a non-response rate of 20% and multiplying by the assumed design effect, 1.5 to adjust variance arising from cluster design, the estimated sample size was 596. We met the principals of the selected ten schools and handed them a request letter from the principal investigator’s institution and a permission letter from DEO for their approval to carry out the study in the respective schools.

### Data collection

After getting permission from the school authority, self-administered questionnaires were distributed to a total of 646 children, with instructions about the questionnaire by the principal investigator and respective class teacher. A separate request letter to the parents, prepared by the school, along with an informed consent paper, was attached to the questionnaire and distributed among the parents through the eligible children. Then, questionnaires filled by parents were collected at the school after two days, and the weight and height measurements of each child were taken using a digital weighing machine and standard measuring tape respectively.

### Study variables

We measured the following characteristics:

### Socio-demographic

Socio-demographic factors included age, sex, birth-weight (categorized as low birth weight: < 2.5 kg; normal weight: 2.5–4.0 kg; and large weight for gestational age: > 4.0 kg), family type (nuclear, joint and extended) and ethnicity (categorized as Brahmin/Chhetri, Janajati, Madhesi, Muslim, Dalit and Others according to Health management information system, Nepal government) [23]. Similarly, socioeconomic characteristics included education level of both father and mother (below high school: < 10th grade; high school: 10–12 grade; and university level: higher education), occupation of both father and mother (unskilled worker, skilled worker, clerical/shop-owner/farmer and profession) and monthly income of the family in NRs (< 10,000, 10,000–25,000, 25,001–50,000 and > 50,000). (102.5 NRs =1 USD on the day of data collection.) All variables were self-reported by a parent of the child.

### Physical activity

We used the Physical Activity Questionnaire for Children (PAQ-C) [24], translated and adapted so it best fits the Nepalese context. The questionnaire, designed for students 8 to 14 years old, contains questions concerning the students’ physical activity in the last 7 days and includes a list of ten items of physical activities (corresponding to Nepali context), each scored from 1 to 5, known as item scores. It has questions about the number of times (frequency) any physical activity is done by a child in the last 7 days and the frequency is given an individual item score as 1 for “no activity”, 2 for “1–2 times”, 3 for “3–4 times”, 4 for “5–6 times” and 5 for “7 times or more” per week. PAQ-C score cutoff points have been proposed to categorize according to their reported PA. The final mean score is obtained by adding all the item-wise scores and dividing by the total number of items. The mean scores 1–5 represent “very sedentary”, “sedentary”, “moderately active”, “active” and “very active” respectively. Further, individuals were classified as “active” (mean score ≥ 3) and “sedentary” (mean score < 3). Likewise, for sedentary behaviors, we used the School Physical Activity and Nutrition Survey (SPANS 2010) questionnaire [25]. It contains a list of seven items about sedentary activities (corresponding to Nepalese context). The total hours spent on each of the sedentary activities on any weekday and weekends were calculated separately. Each sedentary activity was scored as 0 for “never”, 1 for “less than an hour”, 2 for “1–3 h” and 3 for “more than 3 hours” respectively for both weekday and weekend. In the Nepalese context, weekdays are from Sunday to Friday and the weekend is on Saturday. A final mean score was calculated by adding all the item-wise scores and dividing by the total number of items. According to the SPANS, the recommended time of sedentary activities is 2 h or less a day. Any sedentary activity done for more than 2 h a day is considered a high sedentary activity.

### Diet

We used the SPANS 2010 questionnaire [25] for assessing the children’s dietary behavior of the last 7 days, translated and adapted so it best fits the Nepalese context. It contains questions on a list of foods and drinks organized by food categories and we asked the respondents (parents) to report how frequently their children usually consumed each of the foods listed. Respondents reported the consumption of fatty meat products, red meat, fried potato products, salty snack foods, confectionery, ice cream and beverages including sugar-sweetened drinks. Item specific sub-scores were calculated which were specified as 0 for never or rarely, 1 for 1–2 times per week, 2 for 3–4 times per week and 3 for more than 5 times per week according to SPANS junk index questionnaire. The mean score was calculated and classified as low junk consumption for less than twice a week and high junk consumption for more than equal to twice a week.

### Weight

Weight was measured without shoes and with minimal clothing using an Omron Model HBF-400 Scale and recorded to the nearest 0.1 pounds. The measurements were taken by the researcher (primary author) herself, in the respective classroom of students during school hours.

### Height

Similarly, height was measured without shoes using a standard tape measure with participants standing against the wall and recorded to the nearest 0.1 cm. The measurements were taken by the researcher (primary author) herself, in the respective classroom of students during school hours.

We pretested the self-administered questionnaire with 60 non-sampled students of a school in Lalitpur (10% of the actual sample size of the study): 30 students each from two primary-level grades. Certain amendments such as simplifying the language, adding physical activity items, explaining sedentary behaviors by giving examples inside bracket, etc. were made in the questionnaire after pretesting.

## Data analyses

We entered the data in Epi-data V.3.1. The anthropometric calculation (Body Mass Index-for-age-sex) was conducted using WHO Anthro plus software V.1.0.4 [26]. The dependent variable of the study was overweight/obesity which was based on the Body Mass Index (BMI) for the age-sex of the children. “Overweight” was defined as having a BMI-for-age between the 85th and 95th percentiles, and “Obesity” was defined as having the BMI-for-age at or above the 95th percentile [3]. Independent variables were socio-demographic factors of children, socio-economic characteristics of respondents, dietary behaviors, physical activity, and sedentary behaviors of the children.

Statistical analysis was performed using SPSS V.21. The prevalence of childhood overweight/obesity and descriptive analysis of the independent variables were reported as proportions. Chi square test and logistic regression were carried out to find the association of variables. Bivariate and multivariate binary logistic regression analyses were conducted to determine the association between dependent and independent variables. Initially, in bivariate analysis, variables were entered one at a time, and unadjusted OR and 95% CI were computed for all independent variables. Multivariate analysis with all independent variables entered at the same time was completed to adjust for the effect of confounding, and adjusted OR and 95% CI were computed.

## Research ethics

We obtained approval from the ethical review board of the Nepal Health Research Council (NHRC) (Ref no. 535). Written permission was taken from the DEO, Lalitpur. Written approval was taken from all the respondent parents and school authorities before data collection. Confidentiality was maintained on personal issues and information about children of the respondents.

## Results

Among 646 distributed questionnaires, 16 students were absent on questionnaire collection day, 30 questionnaires were not responded to and 25 were incomplete. Thus, 575 questionnaires were considered for analysis.

Table 1 shows the demographic and socio-economic characteristics of the respondent parents and the children. Out of 575 respondent parents, 55% were fathers and 45% were mothers. The mean age of the respondent parent (either mother or father) was 37.5 ± 7.5 years. The mean age of the children was 9.0 ± 1.0 years. Among the children, 57% were males and 43% were females. Three-fifths of them belonged to a nuclear family. More than half of the respondent-fathers were professionals. About 52% of the fathers had attained university-level education. Among the respondent mothers, 57% were either unskilled workers or housewives while 28.5% were professionals. Nearly two-fifths of the respondents had a family income of more than 50,000 NRs per month.

**Table 1:** Demographic and socio-economic characteristics of the respondent parents and the children *n* = 575

Characteristics	Frequency (*n*)	Percentage (%)
Respondent Parents		
Gender		
Male	316	55
Female	259	45
Age (in years)		
≤ 29	65	11.3
30–39	290	50.4
40–49	196	34.1
≥ 50	24	4.2
Mean ± (SD)	37.5 ± 7.55	
Father’s education		
Below high school	147	25.6
High school	127	22.1
University	298	51.8
Father’s occupation		
Unskilled worker	20	3.5
Skilled worker	116	20.2
Clerical, shop-owner, farmer	117	20.3
Profession	322	56
Mother’s education		
Below high school	187	32.5
High school	138	24
University	230	40
Mother’s occupation		
Unskilled worker or housewife	328	57
Skilled worker	48	8.3
Clerical, shop-owner, farmer	35	6.1
Profession	164	28.5
Monthly family income (In NRs) (1 USD = 102.5 NRs)		
< 10,000	37	6.4
10,000-25,000	127	22.1
25,001-50,000	204	35.5
> 50,000	207	36
Characteristics of students		
Age		
6–9 years	314	54.6
10–13 years	261	45.4
Mean ± SD	9.31 ± 1.02	
Sex		
Male	328	57
Female	247	43
Birth weight		
Low birth weight (< 2.5 kg)	39	6.8
Normal weight (2.5–4 kg)	512	89
Large weight for gestational age (> 4 kg)	24	4.2
Mean ± (SD)	3.10 ± 0.52	
Family type		
Nuclear	348	60.5
Joint	161	28
Extended	66	11.5
Ethnicity		
Brahmin/ Chhetri	206	35.8
Janajati	245	42.6
Madhesi	117	20.3
Others	7	1.3

Table 2 illustrates the dietary characteristics of children. 1 in every 5 children consumed hot chips, fries, and potato crisps 3–4 times a week. About 17% of them ate confectionaries and ice cream more than 5 times a week. Nearly, 83% of the children took homemade food to school. Likewise, more than 56% drank more than 250 ml of soft drinks a week.

**Table 2:** Dietary characteristics of children *n* = 575

Characteristics		Frequency (*n*)	Percentage (%)
Food items consumed Per week			
Processed meat (sausages, hamburgers, chicken nuggets, roasts)	Never/rarely	150	26.1
	1–2 times	301	52.3
	3–4 times	98	17
	> 5 times	26	4.5
Hot chips, fries, potato crisps	Never/rarely	121	21
	1–2 times	271	47.1
	3–4 times	127	22.1
	> 5 times	56	9.7
Confectionaries (sweets, cakes, donuts, chocolates, and ice-creams)	Never/ rarely	59	10.3
	1–2 times	240	41.7
	3–4 times	179	31.1
	> 5 times	97	16.9
Bring homemade food to school			
Yes		478	83.1
No		97	16.9
Use of soft drinks			
Yes		478	83.1
No		97	16.9
Less than a bottle (< 250 ml)/week		208	43.5
More than a bottle (> 250 ml)/week		270	56.5

Table 3 presents the sedentary characteristics of children. More than half of the children were engaged in small screen recreation (SSR) that includes watching television/DVDs, playing computer games and using cell phones, iPads, etc. for less than an hour a day during weekdays (Sunday to Friday). Almost 33% watched TV for more than 3 h on the weekend. The most preferred sedentary activities of the children on both weekdays and weekend were watching TV, followed by using mobiles, iPads, etc. and playing computer/online games.

**Table 3:** Sedentary characteristics of children *n* = 575

Characteristics		Frequency (n)		Percentage (%)	
Sedentary behavior	Per week	Any weekday	Weekend	Any weekday	Weekend
Viewing television	No	41	83	7.1	14.4
	< 1 h	302	85	52.5	14.8
	1–3 h	187	218	32.5	37.9
	> 3 h	45	189	7.8	32.9
Playing computer/online games	No	146	196	25.4	34.1
	< 1 h	321	157	55.8	27.3
	1–3 h	87	152	15.1	26.4
	> 3 h	21	70	3.7	12.2
Using hand-held devices (mobiles, i-pads, i-pods)	No	100	170	17.4	29.6
	< 1 h	300	157	52.2	27.3
	1–3 h	138	157	24	27.3
	> 3 h	37	31	6.4	15.8
Doing crafts or hobbies (paper crafts, painting, sketching, etc.)	No	157	232	27.3	40.3
	< 1 h	281	196	48.9	34.1
	1–3 h	105	108	18.3	18.8
	> 3 h	32	39	5.6	6.8
Reading for fun	No	139	252	24.2	43.8
	< 1 h	314	215	54.6	37.4
	1–3 h	98	90	17	15.7
	> 3 h	24	18	4.2	3.1
Sitting around (chatting with friends/ on the phone/ chilling)	No	280	335	48.7	58.3
	< 1 h	246	173	42.8	30.1
	1–3 h	39	55	6.8	9.6
	> 3 h	10	12	1.2	2.1

### Physical activity among children

**Fig. 1:**
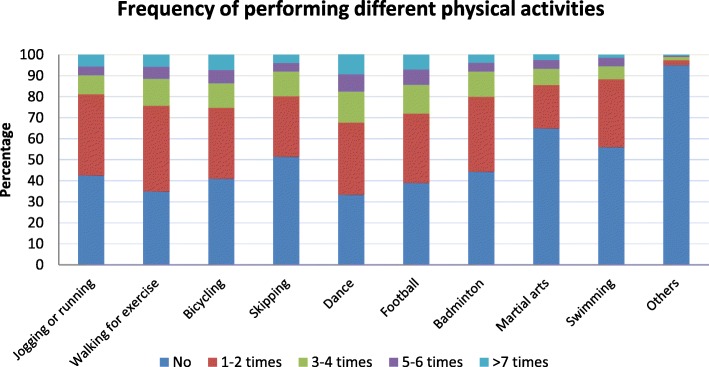
Frequency of performing any form of physical activity

Figure 1 displays the frequency and types of physical activities performed in the last seven days. Dancing, walking for exercise, and bicycling were the most frequently performed activities. On average, at least 50.1% of the male children and 43.6% of female children performed any one form of physical activity at least once a week.

As mentioned in the Methods and Materials section above, individuals are classified as active or having high physical activity if their final mean score is ≥3. Similarly, if their final mean score is < 3, they are considered as sedentary or having low physical activity. In this study, only 3.5% of the study population was found to be having high physical activity, while the rest of the children were found to be having low physical activity.

### Nutritional status of children

**Fig. 2:**
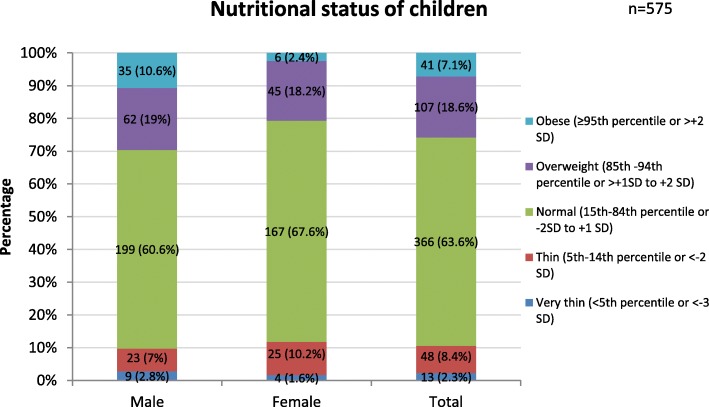
Nutritional status of 1–5 grade children of private schools in Lalitpur district

The prevalence of overweight/obesity among the children was 25.7% (95% CI: 22.1–29.2) as shown in the Fig. 2 . Out of 575 children, 18.6% (95% CI: 15.4–21.8) were overweight and 7.1% (95% CI: 5.0–9.2) were obese. OW/OB was more prevalent among male children as compared to females. About 11% of the children were underweight.

### Factors associated with childhood overweight/obesity

Several factors were found associated with childhood OW/OB in the bivariate analysis as shown in Table 4. Multivariate logistic regressions showed that the sex of children, education level of mothers, high junk food consumption, mode of transport to and from school, and sedentary behaviors on weekend were significantly associated with childhood OW/OB as shown in the table. Male children had double the risk of being OW/OB than female children (aOR = 2.2, 95% CI: 1.3–3.5). Children of mothers who had completed university-level education were 3 times more likely to be OW/OB (aOR = 3.1, 95% CI: 1.2–7.7) than those who had completed lower than high school level. Children who consumed processed meat and its products, (such as sausages, ham, roasts) snacks, (such as potato crisps, chips) and confectionaries (such as sweets and ice cream) more than twice a week were almost 3 times more likely to be overweight or obese than those who consumed less than twice a week (aOR = 2.9, 95% CI: 1.6–5.1).

**Table 4:** Factors associated with childhood overweight/obesity *n* = 575

Characteristics	Overweight/obesity (row %)	Unadjusted odds ratio (95% CI)	Adjusted odds ratio (95% CI)
Related to respondent			
Age (in years)			
≤ 29	14 (21.5)		
30–39	73 (25.2)	1.22 (0.64–2.34)	0.85 (0–38-1.87)
40–49	54 (27.6)	1.38 (0.71–2.71)	0.75 (0.30–1.82)
≥ 50	7 (29.2)	1.50 (0.52–4.33)	0.95 (0.24–3.74)
Sex			
Male	77 (24.4)		
Female	71 (27.4)	1.17 (0.81–1.71)	1.10 (0.65–1.87)
Father’s educational status			
Below high school	22 (15)		
High school	24 (18.9)	1.32 (0.70–2.49)	0.80 (0.32–2.01)
University level	102 (34.2)	2.95 (1.77–4.93)**	0.86 (0.34–2.26)
Occupation of father			
Unskilled worker	7 (35)		
Skilled worker	18 (15.5)	0.34 (0.12–0.97)	0.15 (0.43–0.53)
Clerical, shop owner, farmer	17 (14.5)	0.31 (0.11–0.91)	0.18 (0.05–0.63)
Profession	106 (32.9)	0.91 (0.35–2.35)	0.25 (0.07–0.84)
Monthly income (NRs) (1 USD = 102.5 NRs)			
< 10,000	7 (18.9)		
10,000-25,000	23 (18.1)	0.94 (.37–2.42)	1.23 (0.36–4.22)
25,001-50,000	35 (17.2)	0.89 (.36–2.18)	0.88 (0.25–3.08)
> 50,000	83 (40.1)	2.86 (1.20–6.83)*	1.45 (0.40–5.20)
Mother’s educational ststus			
Below high school	22 (11.8)		
High school	34 (24.6)	2.45 (1.35–4.42)*	3.13 (1.39–7.12)*
University level	87 (37.8)	4.56 (2.71–7.66)*	3.09 (1.23–7.70)*
Occupation of mother			
Unskilled worker or housewife	72 (22)		
Skilled worker	8 (16.7)	0.71 (.31–1.58)	0.90 (0.25–2.25)
Clerical, shop owner, farmer	3 (8.6)	0.33 (.09–1.12)	0.44 (0.11–1.65)
Profession	65 (39.6)	2.33 (1.55–3.51)**	1.34 (1.02–4.05)**
Related to child			
Age (in years)			
6–9	68 (21.7)		
10–13	80 (30.7)	1.59 (1.09–2.32)*	1.36 (0.86–2.16)
Sex			
Male	97 (29.6)	1.61 (1.09–2.38)*	2.21 (1.38–3.53)**
Female	51 (20.6)		
Birth weight			
Normal	132 (24)		
LGA	16 (66.7)	6.34 (2.65–15.16)**	2.42 (0.83–7.01)
Family type			
Nuclear	99 (28.4)		
Joint	38 (23.6)	0.77 (0.51–1.19)	0.77 (0.46–1.28)
Extended	11 (16.7)	0.50 (0.25–1.01)	0.59 (0.27–1.29)
Ethnicity			
Janajati	67 (27.3)		
Not Janajati	81 (24.5)	0.86 (0.59–1.26)	0.78 (0.49–1.25)
Dietary characteristics of children			
Low junk food consumption (< 2 times /week)	100 (21.2)		
High junk food consumption(≥ 2 times/week)	48 (46.2)	3.18 (2.03–4.95)**	2.92 (1.66–5.12)**
Homemade food, taken to school			
Yes	113 (23.6)		
No	35 (36.1)	1.82 (1.14–2.90)*	1.58 (1.11–2.58)*
Drinks soft drinks			
No	25 (25.8)		
Yes	123 (25.7)	0.99 (0.61–1.64)	1.13 (0.63–2.05)
Physical activity level			
Low	148 (26.7)		
High	0 (0)	0	0
Mode of transport to school			
On foot	18 (11.7)		
By school bus	110 (31.8)	3.52 (2.05–6.05)**	2.38 (1.12–4.79)*
Own or public vehicles	20 (26.7)	2.75 (1.35–5.58)**	2.49 (1.24–6.72)*
Sedentary behavior on weekdays			
Recommended (≤2 h a day)	146 (25.7)		
High (> 2 h a day)	2 (33.3)	1.41 (0.26–7.99)	3.2 (0.41–26.90)
Sedentary behavior on weekend			
Recommended (≤2 h a day)	130 (23.9)		
High (> 2 h a day)	18 (56.3)	4.09 (1.97–8.44)**	3.01 (1.20–7.29)*

Hosmer-Lemmeshow goodness of fit test of final model: 0.633

Bold* indicates significance at *p*-value < 0.05

Bold** indicates significance at *p*-value < 0.001(LGA: Large for gestational age)

Children who used school buses (aOR = 2.3, 95% CI: 1.1–4.7) or own or public vehicle (aOR = 2.4, 95% CI: 1.2–6.7) as their primary means of transport to and from school were at twice the risk of being OW/OB than those who traveled on foot. A significant association was not seen between sedentary behaviors on weekdays and childhood OW/OB, but on weekends and childhood OW/OB. Children who exceeded the recommended screen time of less than equal to 2 h a day on weekends were 3 times more likely to be OW/OB than those who met the recommendation (aOR = 3.0, 95% CI: 1.2–7.3).

Also, upon studying association between monthly income of the family and junk food consumption (data not in the table above), it was found that children belonging to families with monthly incomes more than 50,000 NRs (> 487.8$) had 1.7 times more likely of having high junk food consumption than those belonging to families with incomes of less than 10,000 NRs (< 97.6 $) per month. No significant association was found between the monthly income of the family and other behaviors like physical activity and sedentary behaviors.

## Discussion

The study found that one in four children (26%) in grades 1–5 at private schools in Lalitpur Metropolitan City were either overweight or obese, which is congruent with the findings of a study carried out in Nepal (25% OW/OB) [20]. Sex of children, education level of mothers, high junk food consumption, mode of transport to and from school and sedentary behaviors on the weekend were significantly associated with childhood OW/OB.

A study on urban school children in North India also showed that the prevalence of overweight and obesity, based on International Obesity Task Force (IOTF) standards, were 24 and 8% respectively in 2008 [27]. Similarly, a population-based cross-sectional study conducted in Pakistan among primary school children in 2011 suggested that 17% of the children were overweight and 7.5% were obese [28]. Therefore, our findings corroborate with findings from other parts of Asia showing that childhood overweight and obesity affects at least one-fourth of the primary grade children.

In this study, male children were found to be 2 times more likely to be overweight or obese than female children. This is in contrast to the findings from similar studies in other countries like India [27], South Africa [29], China [30], Brazil [31], Pakistan [32], and Vietnam [33] which reported a higher prevalence of obesity in females than in males. This may be due to the preference of a male child in Nepalese society, resulting in male children having fewer siblings than female children. Having fewer siblings has been shown to increase the odds of being overweight or obese in other studies [34, 20]. Being the youngest or only child has also been shown to increase the odds of being overweight or obese [20, 34, 35].).

Children of mothers with university-level education were found to be 3 times more at odds of being OW/OB than of mothers with education below high school, which was supported by a study in India [36]. In Nepal, mothers are the primary caretaker of the children. Educated mothers have a higher chance of employment resulting in lesser time to monitor their children’s physical activities or sedentary behaviors like watching TV, which in turn, significantly increases their BMI [37]. Similarly, children of mothers whose occupation was profession work were 1.3 times more likely to be OW/OB than of mothers who were unskilled workers or housewives. Mothers, although generally being the caretaker of the children, lacking time to have a close look at their children’s food consumption behavior, physical activity, and sedentary behaviors due to their occupation roles might be attributed to the fact that their children are OW/OB [38]. Also, working mothers that can afford junk food for their children may cook fewer meals at home, thus opting for more restaurant meals and fast foods that are densely packed with calories, therefore resulting in OW/OB [37].

About 18% of the children qualified for high junk food consumption (≥ 2 times a week) and almost half of them were overweight/obese. Children who consumed the specified junk foods in the study twice or more in a week were 2.9 times more likely to be OW/OB than those who consumed less than twice a week. This is because junk food, with high levels of saturated fats, sodium, and sugar, has major links to causing obesity children [39, 40].

Children belonging to families with monthly income more than 50,000 NRs (> 487.8$) were 1.7 times more likely of having high junk food consumption than those belonging to families with income of less than 10,000 NRs (< 97.6 $) per month. This could be because a higher income status could lead to unrestricted access to energy dense fast foods in the family, luxurious lifestyle, and high daily expenses to children. Children who ate non-homemade food at school were 1.5 times more likely to be OW/OB than who took homemade food to school. This could be due to easy access and consumption of high-calorie nutritionally depleted foods in school cafeterias and fast-food stops located in the school neighborhood. This was also supported by studies in other developing nations [41–43].

No significant association was found between high versus low physical activity and OW/OB. It was contradictory to other studies that showed a significant association between physical activity and childhood OW/OB [43–45]. This result may be because of certain limitations that are inherent to the use of self-administered questionnaires such as the exclusion of some items from the PAQ C irrelevant to this study, like activity during physical education classes, at recess, etc. That could have affected the mean PAQ score and hence the association with BMI.

However, the finding that 96.5% of the children had low physical activity and only 3.5% had a high physical activity level is congruent to a similar study reported by Rivera et al. [46].

Children who used school buses were 2 times more likely of being OW/OB than active commuters. This might be due to higher energy expenditure while walking thus creating a protective factor for OW/OB, which is congruous to previous studies done in similar settings [47, 48].

Only 1% of the children had high sedentary behavior (> 2 h a day) on any weekday whereas, almost 6% of them had high sedentary behavior on the weekend. Those children were 3 times more likely to be OW/OB than those having a recommended sedentary behavior of less than equal to 2 h a day on the weekend. These findings were supported by findings from previous studies in similar settings [49–52]. This fact may be associated with a lack of parental control over this sedentary habit, which often causes children to demand candies and sweets advertised on TV [53].

### Strengths and limitations

This study has several strengths. The weight and height of children were measured objectively. Schools and study participants have been selected randomly. The sample size is reasonably large (*n* = 575) and the study can be representative of urban Lalitpur and similar settings in urban Nepal. Also, this is a novel study regarding children’s overweight and obesity and its associated factors representing primary school children of urban Nepal since previous studies in Nepal have mainly focused on socio-demographic factors related to childhood overweight/obesity (OW/OB). Limited data is available on diet and activity-related behaviors associated with childhood OW/OB.

This study has some limitations as well. Due to the cross-sectional nature of the study, a causal relationship cannot be established. However, the study presents a list of probable risk factors that could be investigated in longitudinal studies in the future. As the study was conducted only in private and primary schools in an urban area of Lalitpur district, it might not reflect the overall scenario of urban Nepal. In addition, the findings may not be generalizable for students in public schools.

The behavioral findings were based on subjective measurements; hence they were likely to have social desirability bias leading to over-reporting of desirable diet or physical activity or under-reporting of undesirable diets and physical activity. Responses were also subject to recall bias (like dietary behaviors: frequency of consumption of meat in the past seven days; physical activity: number of times any physical activity was performed in the past seven days etc.). Thus, the findings need to be interpreted cautiously. This study explained that high junk food consumption and high sedentary activity were significantly associated with childhood overweight/obesity. However, we did not find a significant association between fruits and vegetable intake and obesity in our study, unlike few studies carried in the past which explained an inverse association between fruits and vegetable intake and obesity [54, 55]. Since PAQ-C is designed to assess general levels of physical activity, it does not provide an estimate of caloric expenditure or specific time and intensity information. Hence, the findings need cautious interpretation. Similarly, the dietary questionnaire used here (SPANS 2010) does not accurately quantify amounts of junk foods consumed therefore estimates of the percentage of students meeting dietary recommendations must be interpreted with caution. Although SPANS questionnaire and PAQ-C have been used in developed and developing countries including Nepal, its validity and reliability have not been examined in Nepal which could be a possible limitation of the study. This might have resulted in the measurement error of physical activity and diet. However, we expect this error to be non-differential. Non-differential measurement error might have led to the underestimation of the odds ratio [56].

## Conclusions

One in every four children of the age group 6–13 years old, going to private schools in Lalitpur, Nepal were either overweight or obese. Male children, children born to mothers with high school level education or above, and to those engaged in professional work, children who consumed junk food, who didn’t take homemade food to school, who lack active commute to school, and who had a high level of sedentary behaviours on weekends were more likely to be OW/OB. Early interventions on these modifiable risk factors are likely to reduce the rate of childhood obesity. School health programs by concerned stakeholders in promoting low intake of junk food, active commuting to school and active lifestyle among children are suggested. Further studies with objective measurements to identify the association between diet-and-activity-related-behaviors and childhood overweight and obesity among Nepali school children are highly recommended.

## Data Availability

All data is available in the paper. The datasets generated during and/or analyzed during the current study are available from the corresponding author on reasonable request.

## Abbreviations

BMI: Body Mass Index
DEO: District Education Office
DoHS: Department of Health Services
GoN: Government of Nepal
IOM: Institute of Medicine
IOTF: International Obesity Task Force
IRB: Institutional Review Board
NDHS: Nepal Demographic and Health Survey
NHRC: Nepal Health Research Council
OW/OB: Overweight/obesity
PAQ C: Physical Activity Questionnaire for Children
SPANS: Schools Physical Activity and Nutrition Survey
